# Multi-physics coupling simulation of electrode induction melting gas atomization for advanced titanium alloys powder preparation

**DOI:** 10.1038/s41598-021-02316-w

**Published:** 2021-11-29

**Authors:** Hailin Li, Yongpeng Shen, Pu Liu, Weihua Liang, Mingjie Wang, Shuhong Wang

**Affiliations:** 1 College of the Electrical and Information Engineering, Zhengzhou University of Light Industry, Zhengzhou, 450000 China; 2 School of Electrical Engineering, Xi’an Jiao Tong University, Xi’an, 710000 China

**Keywords:** Engineering, Electrical and electronic engineering

## Abstract

A numerical modeling method is proposed for the melting process of Titanium metals of Titanium alloys powder preparation used for 3D printing. The melting process simulation, which involves the tight coupling between electromagnetic field, thermal field and fluid flow as well as deformation associated during the melting process, is conducted by adopting the finite element method. A two-way coupling strategy is used to include the interactions between these fields by incorporating the material properties dependent on temperature and the coupling terms. In addition, heat radiation and phase change are also considered in this paper. The arbitrary Lagrangian–Eulerian formulation is exploited to model the deformation of Titanium metal during the melting process. The distribution of electromagnetic flux density, eddy current density, temperature, and fluid flow velocity at different time can be determined by utilizing this numerical method. In a word, the method proposed in this paper provides a general way to predict the melting process of electrode induction melting gas atomization (EIGA) dynamically, and it also could be used as a reference for the design and optimization of EIGA.

## Introduction

3D printing, also known as additive manufacturing, offers unrivaled design freedom with the ability to manufacture components from a wide range of materials. It is a technology that produces 3D parts layer by layer from materials, such as polymer and metal. 3D printing offers a possibility to produce complex components without design constraints from the traditional manufacturing routes^1^. Powder preparation is one of the key issues for 3D printing. For 3D printing of the end product of the metallic parts, the characteristics of metal powders and the processing of 3D printing, such as sintering, infiltration and surface finishing etc., determine their performances^2^.

Material properties of the metal powder will affect the surface quality of 3D printing parts. To guarantee the precision of the metal component production, the metal powders should have consistent characteristics^3^. The primary characteristics of metal powders are their size distribution and morphology. Other characteristics include density, sintering ability, chemical composition, surface area, thermal properties, and so on^4^. Generally, the powder preparation methods of spherical metal powders for 3D printing can be classified into different categories, such as gas atomization, plasma gas atomization, electrode induction melting gas atomization (EIGA), induction melting, water atomization, and vacuum induction melting^2,5,6^. Because of its low cost, high efficiency, high quality, easy control, and mass production potentiality, the gas atomization technique is widely used^7^.

EIGA, as an alternative, ceramic-free melting method in place of the conventional directly contacting melting method, has the characteristics of high efficiency, large output, and small powder size^8^. Hence, it is especially suited for the preparation of high-purity, refractory and reactive metal powders, such as Titanium, Niobium, and Zirconium. Numerical modeling of the melting process is helpful for a better understanding of the design of EIGA. For example, it could help to deepen understandings of the details of inductive coupling and favorable melting conditions. What is more, it also helps to determine what kind of nozzle should be used for gas atomization, and it is beneficial for the control of speed gas stream injecting into the nozzle^9^. Therefore, it is necessary to study the numerical simulation of the EIGA melting process. Besides that, it avoids a large number of the EIGA prototype manufacturing for validation and saves the cost.

The melting process of EIGA is very complex. It involves multi-physics interactions, such as electromagnetic, heat transfer, and fluid flow fields as well as shape deformation of the melting metal during the whole melting phase. In other words, it is a two-way tight coupling problem involving different physics fields.

Many researchers have performed a series of works to study the melting process of EIGA. Bojarevics proposed a numerical model of electrode induction melting process for EIGA and investigated the complex interactions of the electromagnetic field, thermal fields and the fluid flow field with a free surface by using the free surface code SPHINX and the commercial software COMSOL^10^. Shan did research on the coupling of the electromagnetic and thermal field of novel induction melting coils for the nickel-based super-alloy^11^. A 2-D axisymmetric model for the molten metal flow with a free surface in an alternate electromagnetic field has been developed by using the coupling between Lorentz force and Joule heat upon the new free surface shape in ANSYS, and the transient two-phase volume of fluid turbulent fluid flow calculation with repeatedly updated heat and momentum sources in FLUENT^12^. Other works related to numerical modeling of inductive heating, such as electromagnetic melting and electromagnetic levitation melting, also have similar phenomena with EIGA. Gagnoud studied the free boundary problem of electromagnetic levitation melting and the continuous casting process^13^. Yoshikawa researched the deformation of the molten metal by using the finite element method and moving particle semi-implicit method to calculate the electromagnetic field and the fluid field, respectively^14^. Kermanpur conducted a numerical simulation of the coupling between electromagnetic field and thermal field, respectively^15^. The melting process of the titanium was performed, and the Oscillations, as well as the dynamic surface of the liquid metal were also simulated by resorting to the arbitrary Lagrangian–Eulerian method^16^.

Nevertheless, these works were not taking the two-way coupling of physics fields into consideration. A sequence coupling strategy was adopted by these works, which neglects the tight coupling effects of these physic-fields involved. What is more, the mesh would be distorted, even inverted, by adopting the Lagrangian method when it comes to large deformations. The arbitrary Lagrangian–Eulerian formulation combines the merits of Eulerian and Lagrangian formulations, and it overcomes their shortcomings at the same time. It has been used to track the shape of fluid flow and electromagnetic levitation melting in many numerical simulations^16,17^. This method is adopted in this paper to track the large deformation of the fluid surface as its shape changes with the resultant force exerted on it.

To the best knowledge of the author, there are few mathematical models for the numerical simulation of the whole melting process involving the two-way coupling of different physics fields, from the initial preheating solid state to the formation of the melting stream, and even droplet at the tip. This paper deals with the numerical modeling of the whole melting process of the EIGA. It is helpful for the predicting of the melting process of EIGA dynamically, and it could be also used as a reference for the design and optimization of EIGA by regulating the parameters involved during the numerical simulation. Besides that, the cost can be saved. Here in this paper, the Titanium metal is used as an example to present the performance of the numerical modeling of EIGA melting. The organization of this paper is briefly introduced. Firstly, the mathematical model for the melting process simulation is put forward in “Mathematical model” section, including the principle of EIGA, material properties, assumptions, and the governing equations for the melting process involving multi-physics coupling. Then, the coupling strategy is introduced in “Coupling strategy for multi-physics modeling” section. Results and discussion is followed in “Results and discussion” section. The conclusion is presented in “Conclusions” section.

## Mathematical model

### Principle of electrode induction melting gas atomization

**Figure 1:**
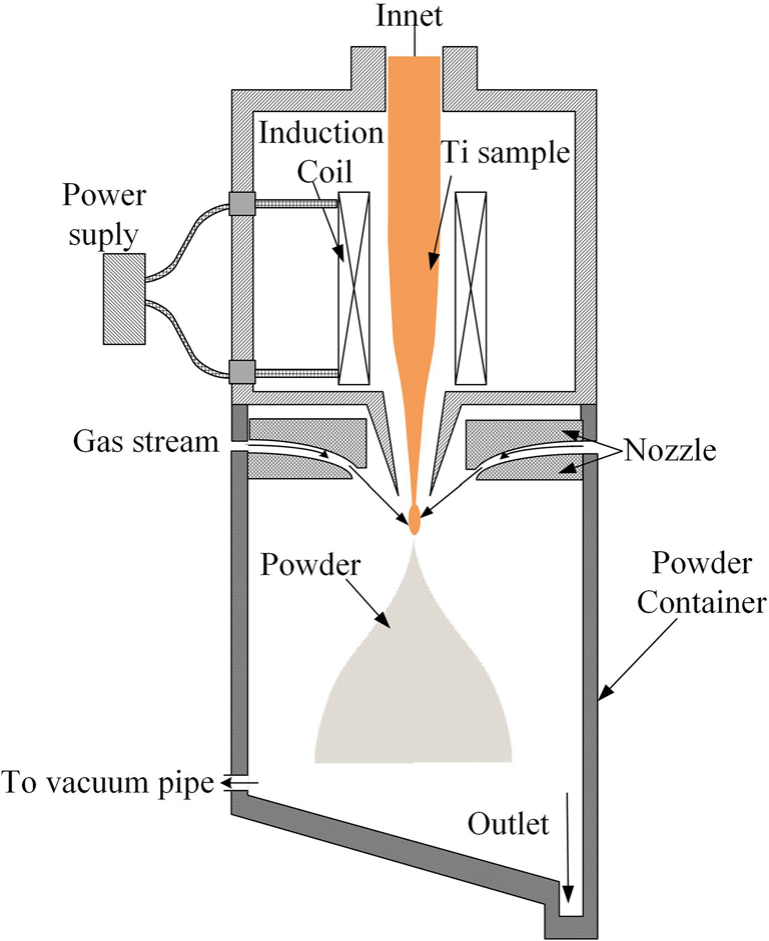
The schematic diagram of the EIGA.

The schematic diagram of the EIGA is illustrated in Fig. 1. As it is shown, this device consists of several components, namely, induction coils, gas nozzle, powder container, and vacuum pipe. The induction coil and gas nozzle are the two most important components. The induction coil is used for metal melting. The nozzle is responsible for the powder preparation of the melting metal by injecting a high-speed gas stream. In order to avoid contamination during the metal powders preparation, it is recommended to use a non-contact melting method and inertial gas, especially for the preparation of reactive, high-purity, and refractory metal powders. Here, the Titanium metal samples are heated by the EIGA to manufacture Titanium alloy powders.

### Material properties and assumptions

#### Material properties

**Figure 2:**
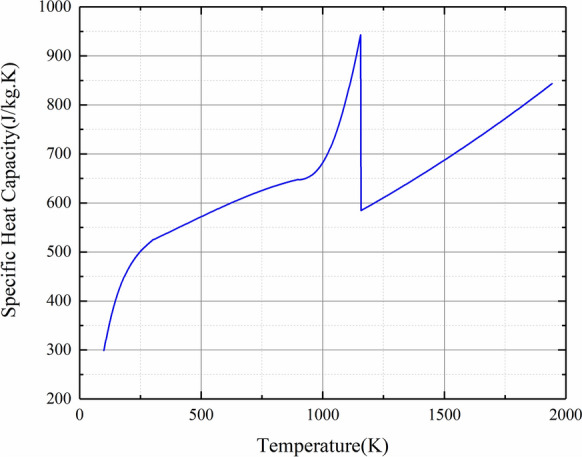
Specific heat capacity versus temperature.

**Figure 3:**
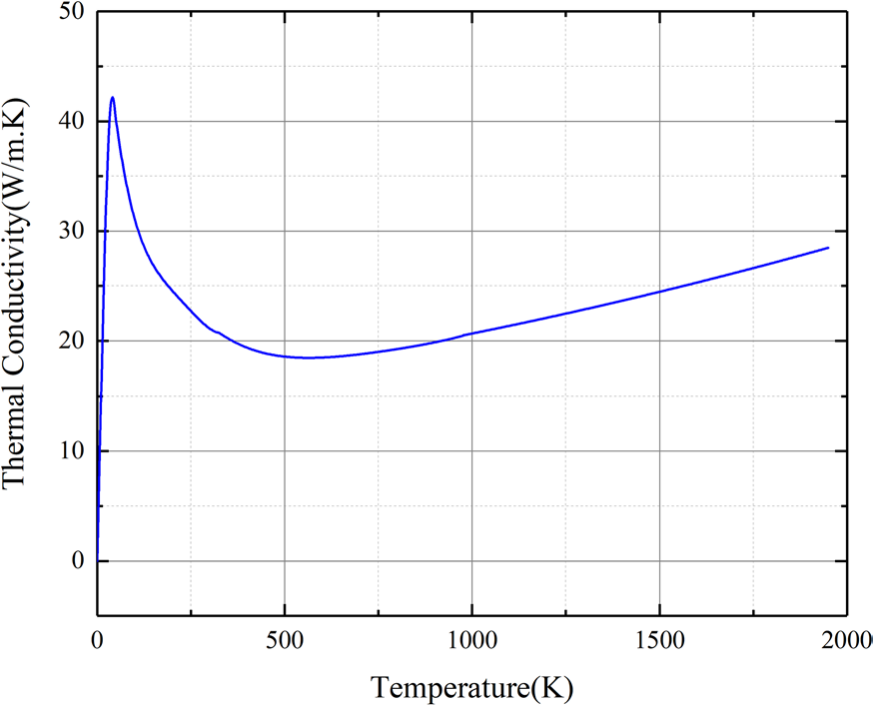
Thermal conductivity versus temperature.

**Figure 4:**
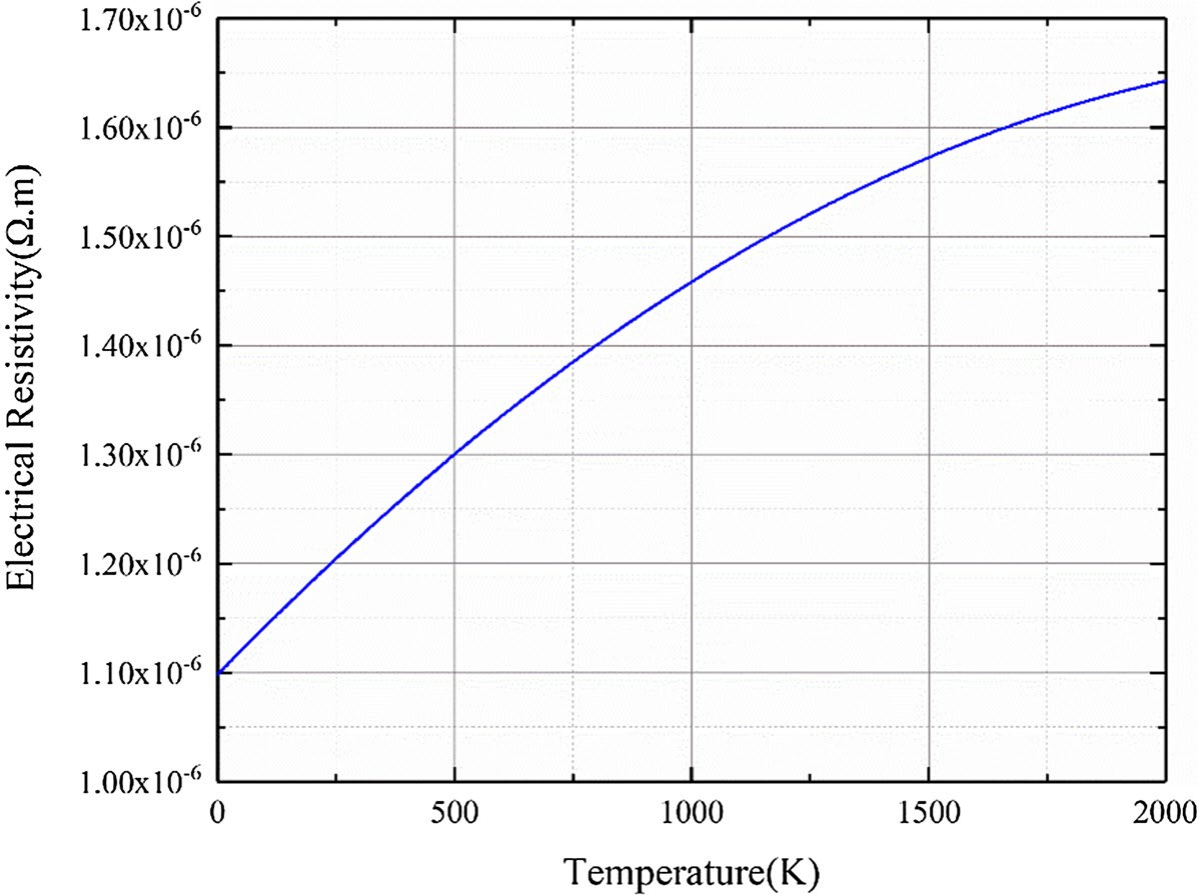
Electrical resistivity versus temperature.

Because of the tight coupling between these physical fields, thermal conductivity, specific heat capacity and electrical conductivity used for simulation are temperature-dependent parameters. The material properties of Titanium changing with temperature are given in Figs. 2, 3 and 4.

#### Assumptions

1. Hollow conductors are used to make the induction coil. Joule heat generated by the electric current can be dissipated by adopting this kind of conductors with cooling water flowing through. The electric current of the induction coil is assumed to be uniformly distributed. The electric eddy current in the coil is not taken into consideration.
2. As it is known, the time constant of the thermal field is typically 10^4^ times larger than the time constant of the electromagnetic field with operating frequency up to tens of kHz. Thus, it would be very time-consuming to solve such a fully-coupled and transient multi-physics coupling system. In order to reduce the simulation time and maintain the computation accuracy to the best, we model the electromagnetic problem as a time-harmonic system weakly coupled to a time-transient non-linear thermal and fluid flow system. The time-step is 0.01 s for both thermal field and fluid flow field.
3. The Titanium to be modeled can be considered as fluid, while the viscosity of its solid-state can be assumed to be infinite ideally. The viscosity is set to be a large but finite value to prevent flow from occurring in the solidified material^10^. Here, this value is set to be 1.0e^7^. As its melting temperature, 1950 K, is reached, the viscosity for its liquid state is determined by its actual material properties. Finally, the viscosity of Titanium used for simulation can be described by the following piece-wise function,
  1 $$\mu = \left\{ {\begin{array}{*{20}l} {80000, } \hfill & {T < 1950K} \hfill \\ {0.06071 - 6.890 \times 10^{ - 5} T + 2.695 \times 10^{ - 8} T^{2} - 3.583 \times 10^{ - 12} T^{3} ,} \hfill & {T \ge 1950K} \hfill \\ \end{array} } \right.$$

**Figure 5:**
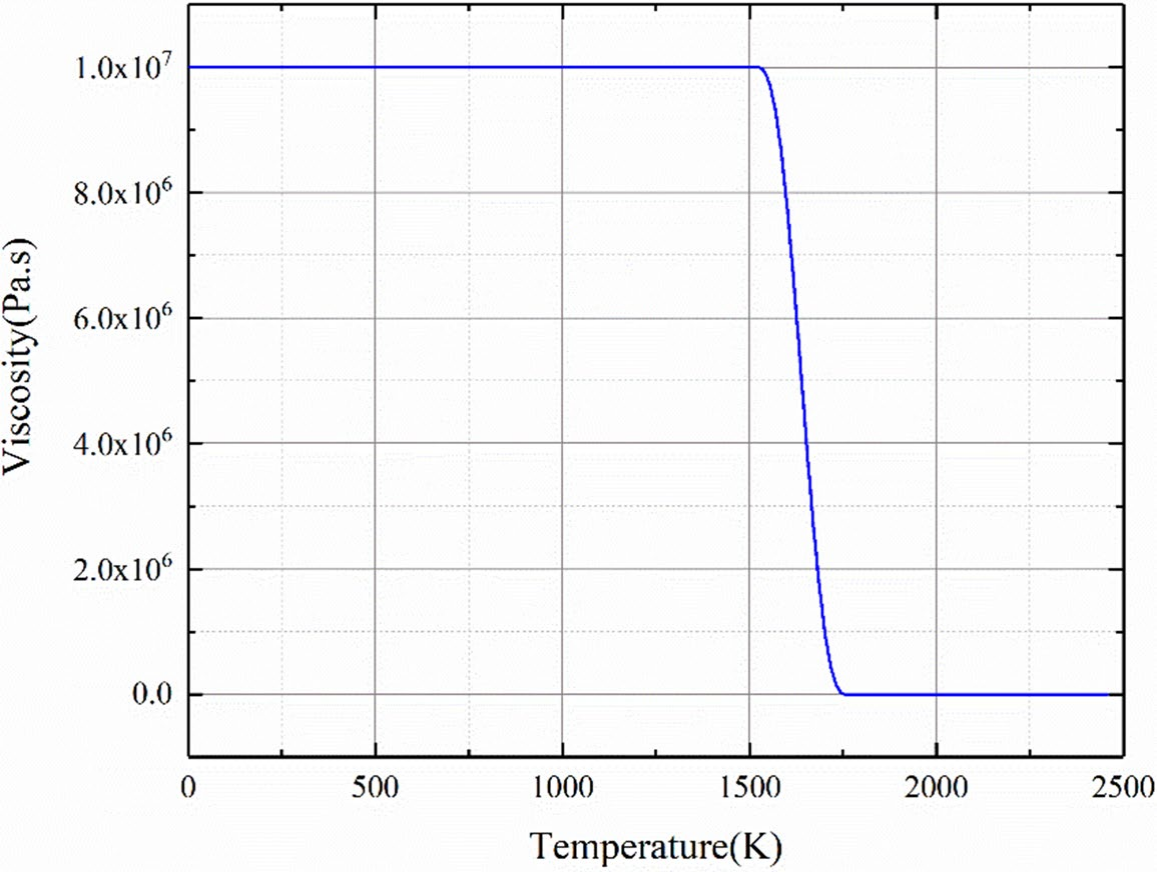
The viscosity of titanium alloys for simulation.

The viscosity curve of Titanium used for simulation in this paper is illustrated in Fig. 5.

### Multiphysics coupling mathematical models for electrode induction melting gas atomization

#### Geometry model

**Figure 6:**
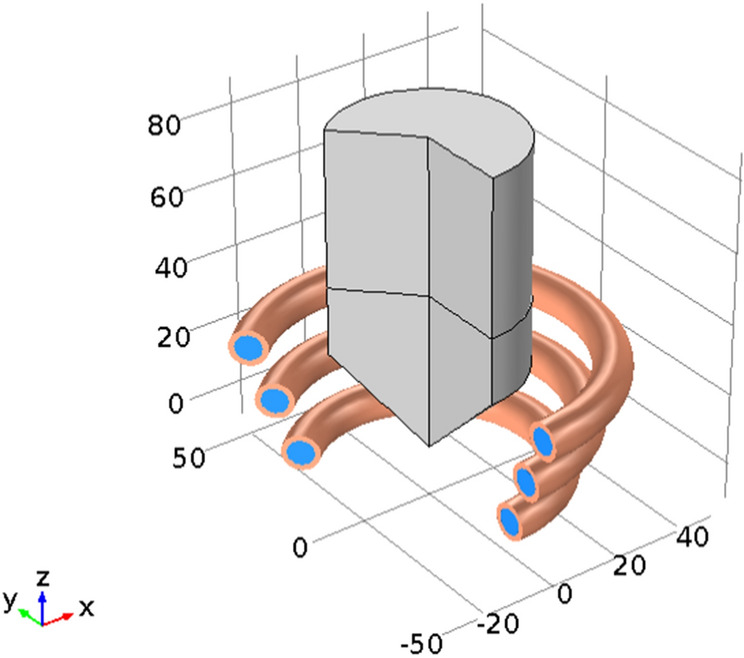
Simplified geometry model of EIGA.

As shown in Fig. 6, a simplified geometry model of EIGA for simulation is demonstrated. A cone-shaped coil is arranged in the bottom of Titanium metal samples, and a Titanium alloy rod is placed in the center place of this coned-shaped coil. The diameter of the rod is 50 mm. The rod bottom is sharpened to an angle of 90°. The bottom diameter of the cone-shaped coil is 54.8 mm, and the diameter for the top turn is 79.7 mm. There are 3 turns for this coned-shaped coil. As mentioned in “Material properties and assumptions” section, the coil is made of a copper tube. The outer diameter of this copper tube is 10.3 mm, while the internal diameter is 7.3 mm. The height of this coned-shape coil is 40.2 mm. The cooling water flowing through the copper tube is adopted to dissipate the heat generated by the Joule heat.

This simplified simulation model can be further simplified into a 2D axisymmetric model because of its 3D axisymmetric geometry structure. Based on this assumption, governing equations for the melting process of EIGA devices can be deduced in the following sub-sections.

#### Magnetic field

For this 2D axisymmetric model, magnetic vector potential in the angular direction exists only, i.e. ***A*** = ***A***_θ_***e***_θ_, ***A***_r_ = ***A***_z_ = 0. In the eddy current region, the governing equation for the time-harmonic electromagnetic field of the EIGA device is

2 $${\upnu }_{0} {\nu }_{\text{em}}\left[\frac{\partial }{\partial z}\left(\frac{1}{r} \frac{\partial \left(r \dot{{A}_{\uptheta }}\right)}{\partial z}\right)+\frac{\partial }{\partial r}\left(\frac{1}{r} \frac{\partial \left(r \dot{{A}_{\uptheta }}\right)}{\partial r}\right)\right]={\text{j}}\omega \sigma \dot{{A}_{\uptheta }}$$

where ν_em_ is the relative magnetic reluctivity, ν_em_ = 1, ν_0_ the vacuum magnetic permeability, σ the electrical conductivity which depends on the temperature, ω the angular frequency, and* J*_θ_ the eddy current density in the angular direction since ***J*** = ***J***_θ_***e***_θ_, *J*_*r*_ = *J*_z_ = 0.

For the non-eddy current region, the governing equation is,

3 $${\upnu }_{0} {\nu }_{\text{em}}\left[\frac{\partial }{\partial z}\left(\frac{1}{r} \frac{\partial \left(r \dot{{A}_{\uptheta }}\right)}{\partial z}\right)+\frac{\partial }{\partial r}\left(\frac{1}{r} \frac{\partial \left(r \dot{{A}_{\uptheta }}\right)}{\partial r}\right)\right]={0}$$

For the current source region,

4 $${\nu }_{0} {\nu }_{\text{em}}\left[\frac{\partial }{\partial z}\left(\frac{1}{r} \frac{\partial \left(r \dot{{A}_{\theta }}\right)}{\partial z}\right)+\frac{\partial }{\partial r}\left(\frac{1}{r} \frac{\partial \left(r \dot{{A}_{\theta }}\right)}{\partial r}\right)\right]=\dot{{J}_{s}}$$

where ***J***_s_ is the current source density of the coils.

#### Heat transfer

The governing equation of heat transfer can be described as,

5 $$\uprho {C}_{p}\frac{\partial T}{\partial t}+\uprho {C}_{p}{\varvec{u}}\cdot \nabla T=\nabla \cdot \left(k\nabla T\right)+q$$

where *ρ* is the mass density of the Titanium alloy, *k* is the thermal conductivity depends on the temperature, ***u*** is the fluid flow velocity vector, *C*_p_ is the modified specific heat capacity which accounts for latent heat, *q* is the heat source term, *T* the temperature.

Instead of adding latent heat in the energy balance equation as the Titanium metal sample reaches its phase-change temperature *T*_pc_, it is assumed that the phase transformation occurs in a temperature interval between *T*_pc_ − Δ*T*/2 and *T*_pc_ + Δ*T*/2. In this interval, the material phase is modeled by a smoothed function to represent the fraction of phase before transition, which is equal to 1 before *T*_pc_ − Δ*T*/2 and to 0 after *T*_pc_ + Δ*T*/2. The energy absorbed at the melting temperature is used to modify the specific heat capacity *C*_p_.

#### Fluid flow

For the fluid flow field simulation, the liquid Titanium could be assumed as the incompressible and Newtonian fluid flow.

The continuity equations,

6 $$\rho \nabla \cdot {\varvec{u}}=0$$

The Navier–Stokes equations,

7 $$\uprho \partial {\varvec{u}}/\partial t+\uprho {\varvec{u}}\cdot \left(\nabla {\varvec{u}}\right)=\nabla \cdot \left\{-p\mathbf{I}+\mu \left[\nabla {\varvec{u}}+{\left(\nabla {\varvec{u}}\right)}^{\text{T}}\right]\right\}+{\varvec{f}}$$

where *µ* is the fluid viscosity changing with temperature, ***I*** is the unit diagonal matrix, ***f*** is the volume force, *p* is the fluid pressure.

The fluid flow field inside liquid Titanium is turbulent flow, and this turbulent flow can be described by the following κ–ε equations,

8 $$\uprho \partial \kappa /\partial t+\uprho {\varvec{u}}\cdot \nabla \kappa =\nabla \cdot \left[\left(\mu +{\mu }_{\text{T}}/{\sigma }_{\upkappa }\right)\nabla \kappa \right]+{P}_{\kappa }-\rho$$

9 $$\uprho \partial\upvarepsilon /\partial t+\uprho {\varvec{u}}\cdot \nabla \varepsilon =\nabla \cdot \left[\left(\mu +{\mu }_{\text{T}}/{\sigma }_{\varepsilon }\right)\nabla \varepsilon \right]+\left(\varepsilon {C}_{\upvarepsilon 1}{P}_{\upkappa }-{C}_{\upvarepsilon 2}\rho {\varepsilon }^{2}\right)/\kappa$$

where

10 $${\mu }_{\text{T}}=\rho {C}_{\upmu }{\kappa }^{2}/\varepsilon$$

11 $${P}_{\kappa }={\mu }_{T}\left\{\nabla {\varvec{u}}:\left[\nabla {\varvec{u}}+{\left(\nabla {\varvec{u}}\right)}^{\mathrm{T}}\right]-2{\left(\nabla \cdot {\varvec{u}}\right)}^{2}/3\right\}-2\rho \kappa \left(\nabla \cdot {\varvec{u}}\right)/3$$

The operator “:” in the above equation is defined as,

12 $${\varvec{a}}:{\varvec{b}}={\sum }_{n}{\sum }_{m}{a}_{nm}{b}_{nm}$$

where κ is the turbulence kinetic energy per unit mass, *ε* is the dissipation rate of the turbulent kinetic energy, and *C*_ε1_, *C*_ε2_, *C*_µ_, *σ*_κ_, *σ*_*ε*_ are the constants, and *C*_ε1_ = 1.44, *C*_ε2_ = 1.92, *C*_µ_ = 0.09, *σ*_κ_ = 1, *σ*_*ε*_ = 1.3.

#### Coupling terms

The coupling terms among different physics fields are summarized in the followings. The molten Titanium would be deformed under the resultant volume force ***f*** of gravity *ρg*, the Lorentz force ***f***_m_. Hence, the volume force ***f*** in Eq. (7) can be described by,

13 $${\varvec{f}}={{\varvec{f}}}_{m}+\rho \mathbf{g}$$

where ***g*** is the gravitational acceleration vector. Here,* g* = 9.81 m/s, along the -*z* direction.

The Lorentz forces ***f***_m_ used in the fluid flow field analysis can be calculated by,

14 $${{\varvec{f}}}_{m}={{\varvec{j}}}_{\uptheta }\times {\varvec{b}}$$

where ***j***_θ_ is the induced electric eddy current, and ***b*** the magnetic flux density.

The Joule heat generated by the electric eddy current in the Titanium alloy sample is the heat source for thermal field analysis, and it can be expressed by,

15 $$q=\upsigma \dot{{{\varvec{j}}}_{\uptheta }^{2}}$$

#### Initial and boundary conditions

Boundary conditions for electromagnetic field

1. Magnetic flux parallel boundary conditions at the external boundaries of the simulation domain,
  16 $${\varvec{n}}\times {\varvec{A}}=0$$
  where ***n*** is the normal vector, ***A*** the magnetic vector potential.
2. Initial and boundary conditions for heat transfer

For the heat transfer analysis, there are 2 types of heat dissipation methods for the surface boundary, namely, surface radiation, natural convection. The boundary conditions for the surface radiation and natural convection are determined by the following equations.

For Surface-ambient radiation,

17 $$-{\varvec{n}}\cdot \left(-k\nabla T\right)={\varepsilon }_{r}{\sigma }_{\text{rad}}\left({T}_{amb}^{4}-{T}^{4}\right)$$

where ε_*r*_ is the surface emissivity, σ_rad_ is the Boltzmann constant, *σ*_rad_ = 5.67 * 10^–8^, and *T*_amb_ is ambient temperature, *T*_amb_ = 293.15 K.

It is assumed that the ambient temperature *T*_amb_ is a fixed value, and the ambient can be considered as a black body which means that the emissivity *ε*_r_ = 1.

For Natural convection,

18 $$-{\varvec{n}}\cdot \left(-k\nabla T\right)=h\left({T}_{amb}-T\right)$$

where *h* is the natural convection coefficient. In this paper, *h* = 5 W/(m2*K).

3. Initial and boundary conditions for fluid flow

By adopting the arbitrary Lagrangian–Eulerian formulation, large shape deformation of molten Titanium alloys can be realized. The deformation of the mesh to the initial shape of the simulation model domain is computed by using Winslow smoothing.

The boundary conditions on the free surface of liquid Titanium metal can be described by the hydrodynamic stress tenso Π. The surface tension is equal to the normal stress component of hydrodynamic stress tensor Π, and the tangential stress component is assumed to be zero^6^. They can be described by the following equations.

For the normal stress component,

19 $${\prod }_{nn}=\gamma K$$

For the tangential stress component,

20 $${\prod }_{nt}=0$$

where γ is the surface tension coefficient, *K* is the local average curvature of the surface.

The boundary condition for fluid flow,

21 $$-p\mathbf{I}+\left({\mu }_{\text{T}}+\mu \right)\left[\nabla {\varvec{u}}+{\left(\nabla {\varvec{u}}\right)}^{\text{T}}\right]-2\kappa \rho \mathbf{I}/3=0$$

Initial conditions for fluid flow field, ***u*** = 0, *p*_0_ = 101 kPa.

#### Excitation

1. Electromagnetic field
  The coned-shape coil is fed with an electric current source with the amplitude of 160A, frequency 50 kHz. There are 3 turns used in this model.
2. Heat transfer
  The copper tube of the cone-shaped coil is cooled by flowing water running through the hollow conductors. Heat transferred by the cooling water every second is^8^,
  22 $${Q}_{c}=\frac{\frac{\mathrm{d}{M}_{\text{w}}}{\mathrm{d}t}{C}_{\text{pw}}\left({T}_{\text{in}}-T\right)}{2\uppi {r}_{\text{w}}{A}_{\text{w}}}$$
  where d*M*_w_/d*t* is the mass flow rate of the cooling water, *T*_in_ the initial temperature of cooling water, *C*_pw_ is the specific heat capacity of cooling water, *r*_w_ is the inner radius of the copper tube, and *A*_w_ is the cross-section area of the cooling water flowing through the copper tube.

Here, d*M*_w_/d*t* = 1 kg/min, *T*_in_ = 293.15 K, *r*_w_ = 3.65 mm, and *A*_w_ = 41.83 mm^2^.

## Coupling strategy for multi-physics modeling

**Figure 7:**
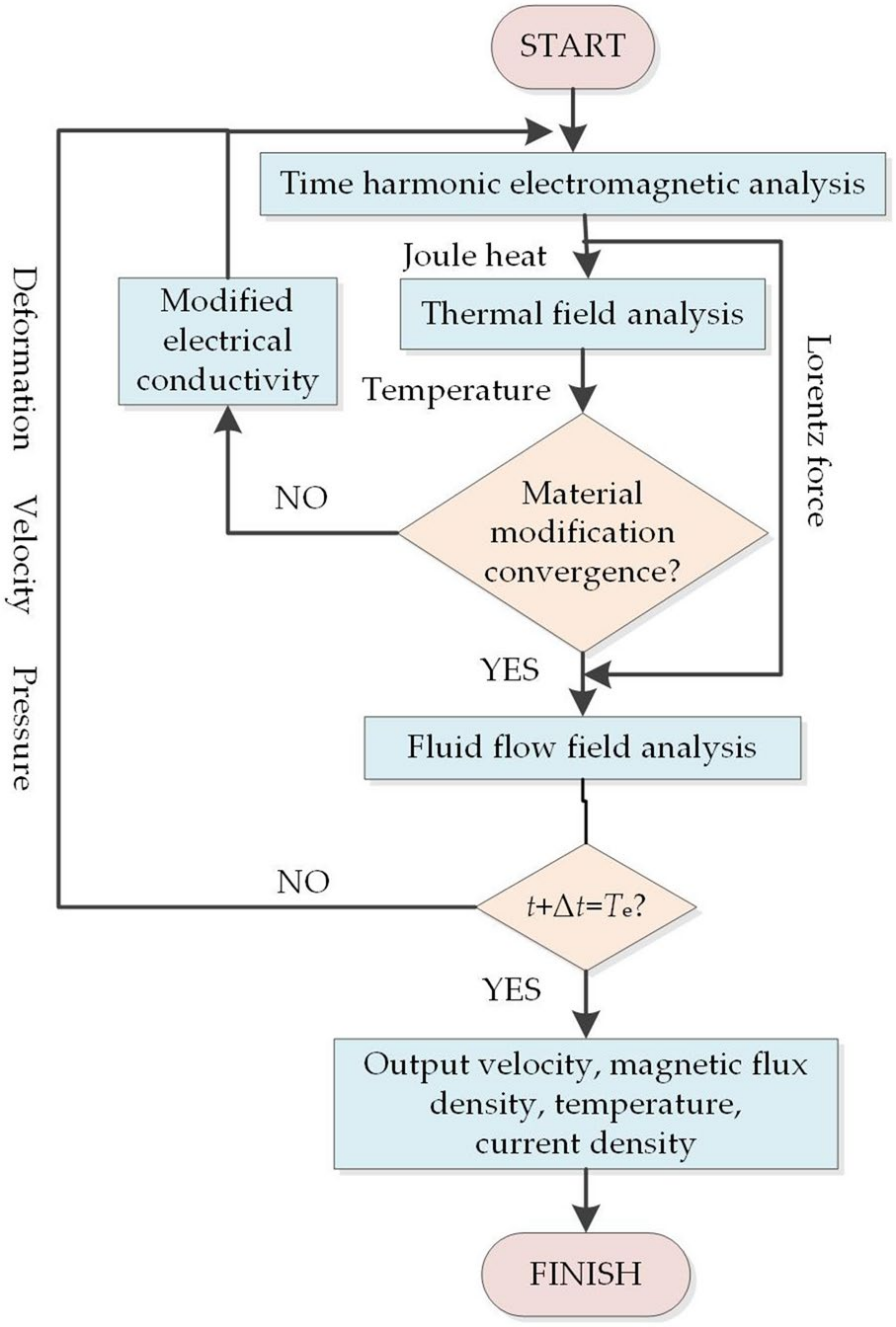
Strategy for multi-physics coupling of EIGA.

As it is mentioned before, the melting process involves the coupling of multi-physics fields. The Titanium alloy is heated by the high-frequency induction coil with the help of Joule heat induced by the electric eddy current. As the increase of temperature of the Titanium alloys, the material properties would change with the temperature, such as the electrical conductivity, dynamic viscosity. In addition, as the Titanium sample reaches its melting temperature, the molten Titanium sample deforms under the influence of electromagnetic force, gravity force, and tensor stress. What is more, this deformation would change the material properties distribution of the original space. In other words, a new geometry model is rebuilt for simulation. Hence, a new meshing is needed during the simulation when deformation happens. This process continues until the simulation time is reached. Because of its complexity involving the two-way coupling of different physics fields, the simulation strategy for this tight coupling should be carefully conducted. The coupling strategy is illustrated in Fig. 7.

Figure 7 shows the way to the coupling simulation of electromagnetic, thermal, and fluid fields. The Joule heat and Lorentz force generated in the harmonic electromagnetic field are transferred to the thermal field and fluid field, respectively. Then, the temperature calculated in the thermal field is feedback to the electromagnetic field to revise the material properties which are dependent on temperature. When the material modification converged, the fluid flow would be driven by the resultant force of Lorentz force and gravity, and the sample would deform. When the time meets the prescribed setting time *T*_e_, the simulation is finished and outputs results of different physics fields. Otherwise, the deformation, velocity and pressure calculated in fluid flow field analysis are passed to the electromagnetic field. The deformation is used to update another round of numerical analysis, while the velocity, and pressure are fixed for thermal analysis. By doing so, the two-way coupling of different physics fields can be realized. This coupling strategy involves the two-way influences of different fields and the revised materials. It is more accurate compared with the weak coupling method without considering two-way interplays. In addition, it is also more efficient than the tight coupling method.

## Results and discussion

**Figure 8:**
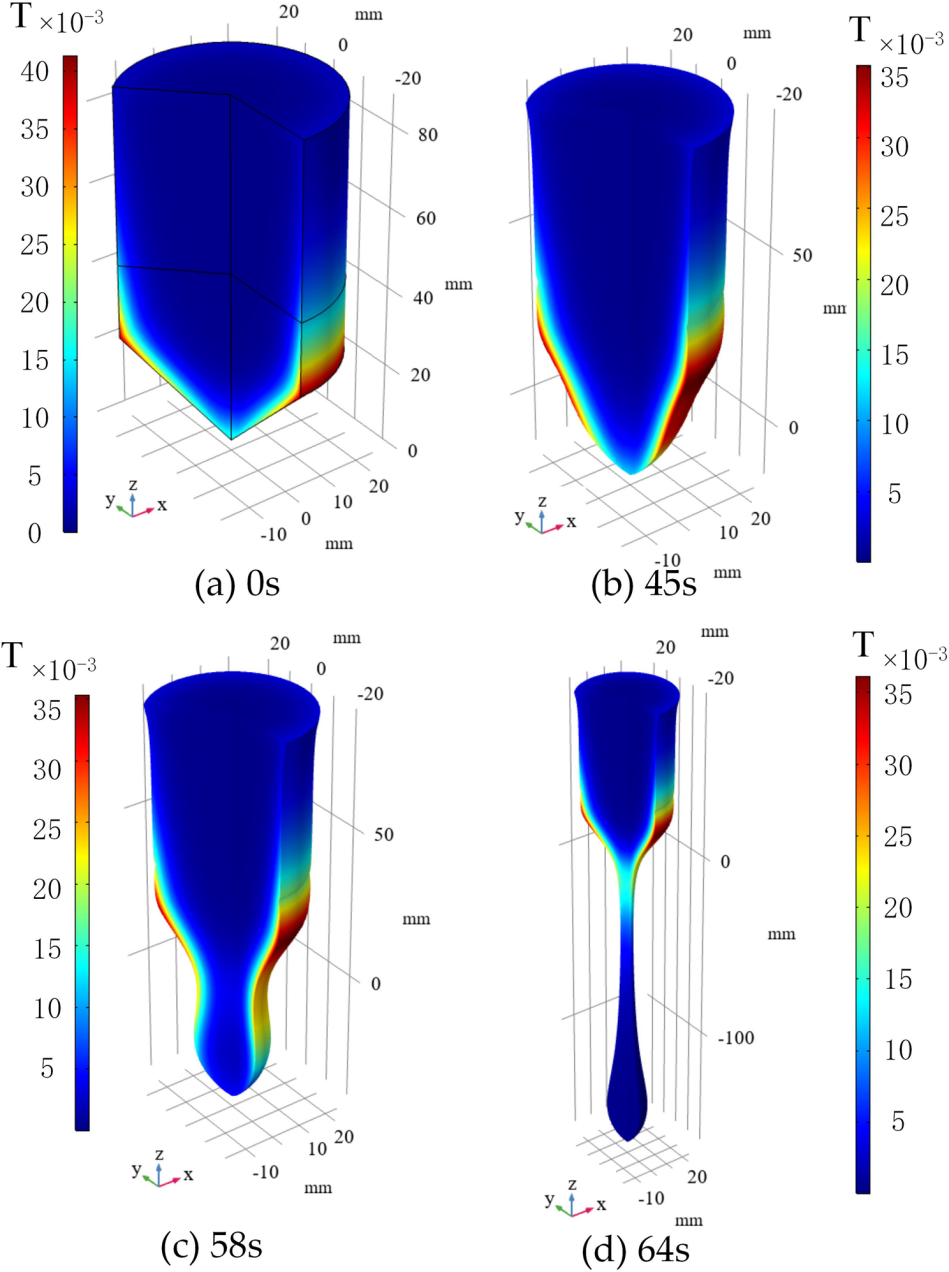
Magnetic flux density distribution of the Titanium sample at different time.

**Figure 9:**
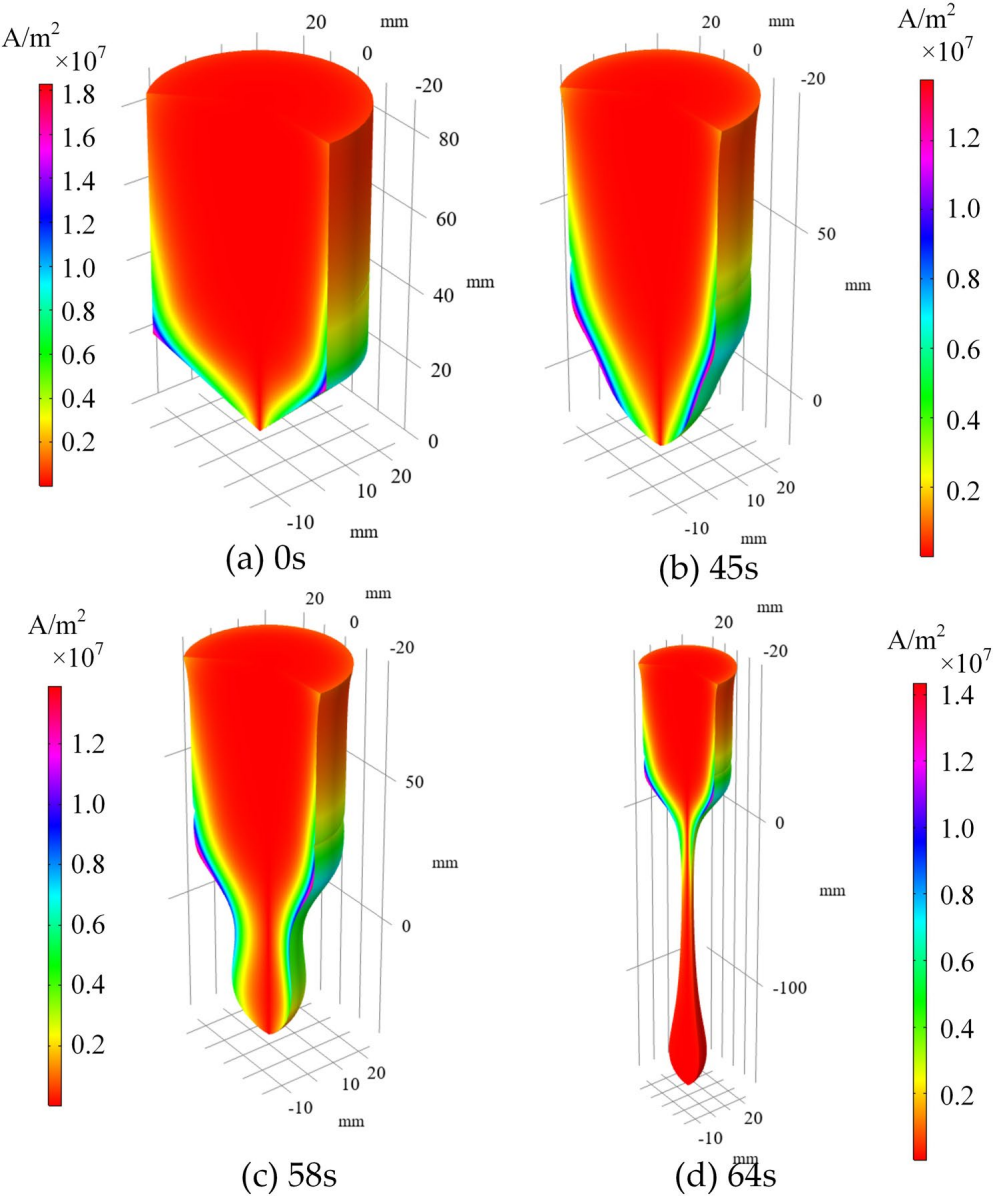
Electric eddy current distribution of the Titanium sample at different time.

**Figure 10:**
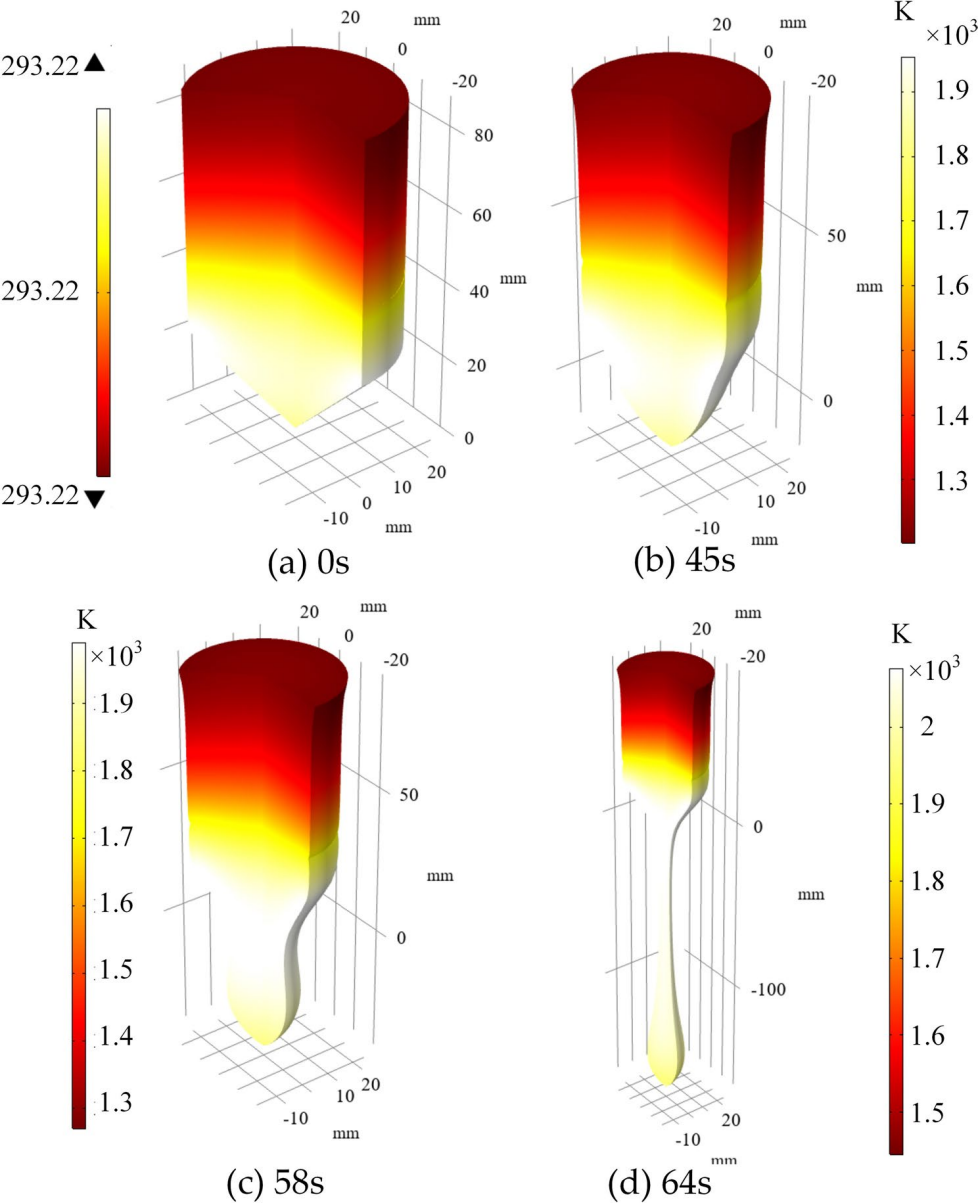
Temperature distribution of the Titanium sample at different time.

**Figure 11:**
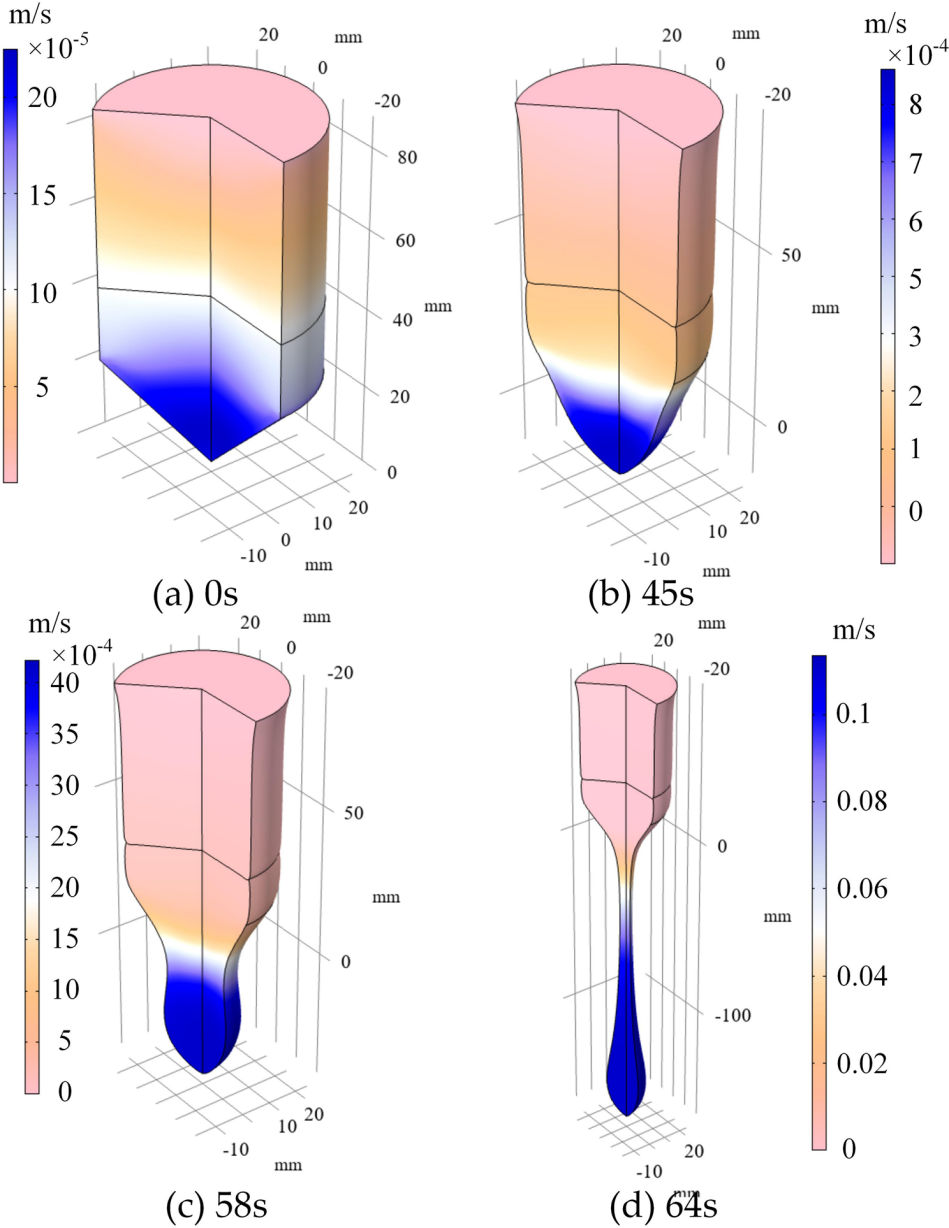
Velocity distribution of the fluid flow field at different time.

The simulation results of EIGA for different fields at certain times are shown in Figs. 8, 9, 10 and 11. Figure 8 is the magnetic flux density at different times during the melting process of a Titanium sample. From this figure, the magnetic flux density distribution changes with the shape of the melting Titanium sample. This phenomenon can be explained by the following. With the increase of the temperature of the Titanium sample, the sample is squeezed by the electromagnetic force so that the material distribution in the original simulation model is changed. These phenomena are clearly seen from Fig. 8a–d. The magnetic flux density is concentrated on the surface of the sample, and the magnetic shield effect occurs which is caused by the high operating frequency of the current excitation of the coil.

The maximum norm value of the flux density is only 0.04 T because there are no ferromagnetic materials used here to collect the magnetic flux density to run through the sample. In such a situation, the coil should better not be far away from the Titanium sample in order to improve the heating efficiency.

The eddy current density induced by the electromagnetic on the sample at different time are given in Fig. 9. As it is known that the eddy current is equal to $-\upsigma \partial A/\partial t$, and ${\varvec{B}}=\nabla \times {\varvec{A}}$. Hence, the eddy current distribution is strongly depended on the distribution of magnetic flux density. Because of the skin effect, the electric eddy current concentrates on the surface of the melting sample. The maximum eddy current density is about 1.83e^7^A/m2, and the maximum value decreased to a certain value, and almost remained unchanged with the deformation occurred. Here the electric eddy current caused by the fluid flow is not considered because the velocity of the fluid flow is too slow to be neglected.

The temperature distribution at different times is shown in Fig. 10. Because of the small thermal conductivity of the Titanium sample, the temperature difference is relatively larger compared with other commonly used metals. The position of the maximum temperature occurred coincides with the position of the maximum eddy current density.

At last, the velocity distribution of the fluid flow is shown in Fig. 11. The speed distribution of fluid flow is the resultant force of electromagnetic force, gravity force as well as surface tension. As the melting sample gets farther away from the coil, the influence of electromagnetic force is getting smaller. Hence, it can be clearly seen from this figure that velocity is getting larger and larger, and gravity dominates. The maximum velocity is only 0.09 m/s. The speed is very slow, and it is the reason for neglecting the electric eddy current induced by the motion of the fluid flow. In addition, because of the existence of surface tension, which has something to do with the surface curvature, the shape of the melting sample is behaved like a droplet. It is believed that this droplet will be separated from the Titanium sample. With the fluid flow speed known, it is helpful for the design of the gas nozzle used for atomization.

## Conclusions

A numerical simulation of EIGA was conducted in this paper to model the truly dynamic melting process of Titanium metals. This simulation involves the two-way coupling of multi-physics fields. With the help of this mathematical model and the coupling strategy proposed in this paper, it could predict the magnetic flux density distribution, eddy current distribution, temperature distribution, and fluid flow velocity distribution of the molten Titanium metal at different times successfully. In a word, the mathematical model could provide a general method for the dynamic predicting of the melting process of EIGA, and it is helpful for the control of the melting process of EIGA. In addition, it is also beneficial for the gas nozzle design of the EIGA, and it is a cost-effective method for the design of EIGA. It could provide a reference for the realization of the true simulation of gas atomization.
